# Ceramides in the Heart: Physiological and Pathological Roles and Regulation

**DOI:** 10.3390/cells15090780

**Published:** 2026-04-25

**Authors:** Xinyi Chen, Oveena Fonseka, Yihua Han, Wei Liu

**Affiliations:** Faculty of Biology, Medicine and Health, The University of Manchester, Manchester M13 9WU, UK; xinyi.chen-2@manchester.ac.uk (X.C.); oveena.fonseka@manchester.ac.uk (O.F.); yihua.han@postgrad.manchester.ac.uk (Y.H.)

**Keywords:** ceramide, sphingolipid metabolism, heart failure, cardiac remodeling, lipotoxicity, mitochondria

## Abstract

**Highlights:**

**What are the main findings?**

- Physiological and pathological roles of ceramides are revealed.
- Ceramides are associated with cardiac dysfunction progression.

**What are the implications of the main findings?**

- Targeting ceramide metabolism may provide potential therapeutic strategies for cardiovascular disease.
- Further elucidation of ceramide signaling regulation may uncover new mechanisms driving cardiac pathology.

**Abstract:**

Ceramides are central bioactive sphingolipids that regulate diverse cellular processes, including membrane organization, energy metabolism, and stress signaling. Emerging evidence has implicated that ceramide dysregulation is associated with the onset and progression of heart failure. This review introduces the understanding of ceramide metabolism, focusing on its biosynthesis, and functional roles in cardiomyocytes. In addition, the contribution of systemic ceramides derived from circulating lipoproteins and peripheral tissues to cardiovascular risk is also discussed. In parallel, it is highlighted that cardiomyocyte-intrinsic ceramide synthesis plays physiological and pathological roles in the heart. Particularly, excessive ceramide accumulation is detrimental for cardiac function, through multiple mechanisms, such as lipotoxic effects, mitochondrial impairment, inflammation, and cell death. The current review discusses the potential diagnostic and therapeutic strategies targeting ceramide metabolism, as well as the open questions about ceramide association with heart disease.

## 1. Introduction

Heart failure (HF) remains a leading cause of morbidity and mortality worldwide, representing the final common outcome of diverse cardiovascular insults including ischemic injury, hypertension, metabolic disease and cardiomyopathies [1,2,3]. Despite substantial advances in pharmacological and device-based therapies, the prognosis of HF remains poor, highlighting the need for improved mechanistic understanding and novel therapeutic targets [4]. In recent years, alterations in cardiac lipid metabolism have emerged as important contributors to myocardial dysfunction, with increasing attention focused on bioactive lipid species that exert regulatory effects beyond their structural roles. Among these, ceramides have gained prominence as key modulators of cellular homeostasis and stress responses [5,6].

Ceramides are central intermediates within the sphingolipid metabolic network and are increasingly recognized as signaling rather than merely structural membrane constituents. Through effects on membrane architecture, intracellular signaling, mitochondrial function, autophagy, inflammation and apoptosis-related pathways [7,8,9], ceramides influence multiple aspects of cellular stress adaptation. Their intracellular abundance is tightly controlled through interconnected pathways of synthesis, degradation, and recycling, allowing dynamic adaptation to metabolic and environmental cues [10].

Importantly, ceramides that influence cardiac physiology and pathology may arise from both systemic and local sources. Systemically, ceramides are produced in peripheral metabolic tissues such as the liver and adipose tissue and circulate in association with lipoproteins, particularly low-density lipoprotein (LDL) and very-low-density lipoprotein (VLDL) [11,12]. In parallel, ceramides are also generated locally within the heart through sphingolipid synthesis and turnover in cardiomyocytes and other resident cardiac cells [13,14,15]. This distinction is especially relevant in HF, where whole-body metabolic disturbance and intrinsic myocardial remodeling frequently coexist and may interact to amplify lipotoxic stress.

Dysregulation of ceramide metabolism has been linked to numerous pathological conditions including insulin resistance, obesity, and cardiovascular disease. In the cardiovascular system, both elevated circulating ceramides and increased myocardial ceramide content have been associated with adverse outcomes. Clinical studies show that specific circulating ceramide species are strongly associated with major adverse cardiovascular events and mortality [16], supporting their value as biomarkers of cardiovascular risk. At the same time, evidence from failing human hearts and experimental models suggests that ceramide accumulation is not simply an epiphenomenon, but may contribute directly to lipotoxic cardiomyopathy, impaired contractile function, mitochondrial dysfunction and susceptibility to cardiac injury [15,17,18].

In this review, we summarize the structural features and biosynthetic pathways of ceramide, their physiological roles in the heart, and the evidence linking ceramide dysregulation to human cardiovascular disease and HF. We also review findings from experimental models, pharmacological and genetic studies addressing causality, and the emerging role of ceramide-induced mitochondrial dysfunction as a mechanistic driver of cardiac injury. Additionally, we discuss potential approaches to therapeutic targeting of ceramide metabolism. Finally, we highlight current understanding limitations of signaling regulation of ceramide metabolism in the heart and insufficient knowledge and its translation into clinical practice.

## 2. Structural Features and Biogenesis of Ceramides

### 2.1. Structural Features of Ceramides

Ceramides are central molecules within the sphingolipid family and serve as the structural backbone from which many complex sphingolipids are derived. Structurally, ceramides consist of a sphingoid base linked via an amide bond to a fatty acyl chain. In mammalian systems, the sphingoid base is highly conserved and is typically an 18-carbon amino alcohol backbone. Structural diversity arises primarily from variation in the N-acyl fatty acid. These fatty acyl chains usually range from approximately C14 to C26 and may differ in both length and degree of saturation, thereby generating a wide spectrum of ceramide molecular species [7,9,19].

The amphipathic nature of ceramides reflects the combination of a smaller polar head group with two hydrophobic chains, namely the long sphingoid base and the N-linked fatty acid. This configuration enables ceramides to integrate efficiently into lipid bilayers and promotes intermolecular packing within membranes. In addition, hydrogen bonding between the amide and hydroxyl groups of neighboring ceramide molecules contributes to the formation of highly ordered membrane domains [19,20,21,22]. These biophysical properties are central to the ability of ceramides to alter membrane organization and to facilitate the assembly of signaling platforms.

Ceramides also act as precursors for a wide range of complex sphingolipids through modification of the primary hydroxyl group at the C1 position of the sphingoid backbone. Among the most abundant derivatives is sphingomyelin, formed by addition of a phosphocholine headgroup. Ceramides can be converted into ceramide phosphoethanolamine (CerPE) and into glycosphingolipids such as glucosylceramide. These complex sphingolipids are subsequently transported through the secretory pathway to cellular membranes, where they become major structural components of the plasma membrane and endomembrane system [10,23]. Thus, the modular architecture of a conserved sphingoid backbone combined with variable acyl chains and headgroup modifications underlies the structural diversity of the sphingolipid family [7,10,19].

### 2.2. Ceramide Biogenesis

**Figure 1:**
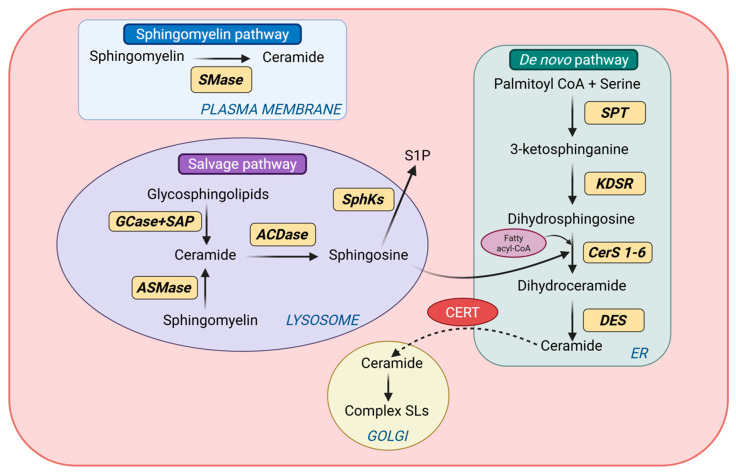
Pathways of ceramide biogenesis and metabolism. Ceramides are generated through three principal pathways within distinct cellular compartments. In the de novo pathway, serine and palmitoyl-CoA are condensed in the ER to form sphingosine, which is subsequently acylated by CerS enzymes using fatty acyl-CoA to produce dihydroceramide. Dihydroceramide is then converted to ceramide by DES. Ceramides can be transported to the GA, where they are converted into complex sphingolipids. Ceramides can also be produced through sphingomyelin hydrolysis by SMases at cellular membranes. In the salvage pathway, complex sphingolipids degraded in lysosomes generate ceramide and sphingosine, which can be recycled to reform ceramide. Abbreviations: ACDase, acid ceramidase; ASMase, acid sphingomyelinase; CerS, ceramide synthase; CERT, ceramide transfer protein; DES, dihydroceramide desaturase; ER, endoplasmic reticulum; GCase, β-glucocerebrosidase; KDSR, 3-ketosphinganine reductase; S1P, sphingosine-1-phosphate; SAP, sphingolipid activator protein; SL, sphingolipid; SMase, sphingomyelinase; SphKs, sphingosine kinase; SPT, serine palmitoyltransferase.

Ceramide biogenesis occurs through three principal routes: (i) de novo synthesis in the endoplasmic reticulum (ER), (ii) hydrolysis of sphingomyelin at cellular membranes, and (iii) salvage and recycling of complex sphingolipids in lysosomes (Figure 1). These pathways are spatially compartmentalized and enzymatically regulated, enabling precise control over ceramide abundance, acyl-chain composition, and subcellular localization. Importantly, because individual ceramide species can exert distinct biological effects, ceramide biogenesis is best understood not simply as bulk lipid production, but as a regulated network that shapes species-specific and compartment-specific signaling [24,25,26].

#### 2.2.1. De Novo Ceramide Synthesis

The de novo pathway is a major source of ceramide production under conditions of nutrient sufficiency and lipid availability. This pathway is initiated on the cytosolic face of the ER by serine palmitoyltransferase (SPT), which catalyzes the condensation of L-serine with palmitoyl-CoA to generate 3-ketosphinganine [27]. SPT functions as a heteromeric complex containing SPTLC1 together with one of the catalytic subunits SPTLC2 or SPTLC3, and this step is widely regarded as the rate-limiting step in sphingolipid synthesis. Subsequent reduction by 3-ketosphinganine reductase (KDSR) yields sphingosine (dihydrosphingosine), which is then N-acylated by ceramide synthases (CerS1–6) to produce dihydroceramides. The final step is catalyzed by dihydroceramide desaturase (DES), which introduces a trans double bond to yield ceramide [28]. Newly synthesized ceramides can then be transported from the endoplasmic reticulum to the Golgi apparatus (GA) via the ceramide transfer protein (CERT) or by vesicular trafficking [29]. A key feature of the de novo pathway is the acyl-chain specificity conferred by the ceramide synthase family [18]. Distinct CerS isoforms preferentially utilizes fatty acyl-CoAs of different chain lengths, thereby shaping the molecular composition of the ceramide pool. For instance, CerS1 predominantly generates C18 ceramides; CerS2 and CerS3 preferentially produces very-long-chain species (C20–C24 and ≥C26, respectively); CerS4 preferentially incorporates C18–C20 fatty acyl chains, and CerS5/6 mainly synthesize C14–C16 ceramides [30,31]. This enzyme-specific chain-length selectivity is important because it provides a mechanistic basis for the functional heterogeneity of ceramide species discussed in later sections. In the heart, CerS2, CerS4 and CerS5 are reported to be particularly abundant under physiological conditions.

#### 2.2.2. Sphingomyelin Hydrolysis

Ceramides can also be generated through hydrolysis of sphingomyelin, a major phospholipid of the plasma membrane and intracellular membranes. This reaction is catalyzed by sphingomyelinases (SMases), which are classified according to pH optima and subcellular location, into acid, neutral, and alkaline isoforms [32]. Acid sphingomyelinases (ASMases) reside primarily within lysosomes but can translocate to the plasma membrane upon stress stimulation. Neutral sphingomyelinases (nSMases), particularly nSMase2, are associated with the plasma membrane and the GA and can be activated by inflammatory cytokines, oxidative stress, and mechanical stimuli. Compared with de novo synthesis, sphingomyelin hydrolysis provides a rapid mechanism for acute ceramide generation in response to cellular stress [32,33,34]. Hydrolysis-derived ceramides promote lateral membrane reorganization through the formation of ceramide-enriched microdomains, which influence receptor clustering and downstream signaling. These localized ceramide pools are therefore functionally distinct from ER-derived ceramides and highlight the importance of spatial compartmentalization in cardiac sphingolipid biology [35].

#### 2.2.3. Salvage and Recycling Pathway

In addition to de novo synthesis and sphingomyelin hydrolysis, ceramides are regenerated through the salvage and recycling pathways, which represents a major route of sphingolipid turnover in cardiomyocytes [36]. Within late endosomes and lysosomes at acidic pH, complex sphingolipids derived from plasma membrane internalization or organelle degradation through autophagic pathways undergo stepwise catabolism mediated by acid hydrolases. Glycosphingolipids are sequentially degraded by exoglycosidases, in cooperation with sphingolipid activator proteins (SAPs), ultimately yielding ceramide as a central intermediate. Lysosomal β-glucocerebrosidase (GCase) contributes to this process by hydrolyzing glucosylceramide to ceramide and glucose, while sphingomyelin delivered to lysosomes can be converted to ceramide by ASMases [37,38]. Ceramides generated in lysosomes may subsequently be hydrolyzed by acid ceramidases (ACDase) to form sphingosine and free fatty acids. Unlike ceramides, sphingosine is sufficiently amphipathic to leave the lysosomal compartment and then can be re-acylated by ceramide synthases at the ER to regenerate ceramide [36,39,40]. Alternatively, sphingosine may be phosphorylated by sphingosine kinases (SphK1 and SphK2) to form sphingosine-1-phosphate (S1P), which is a bioactive lipid with signaling properties often distinct from those of ceramide [41]. Through this recycling system, cells gain metabolic flexibility to rapidly reshape sphingolipid pools in response to stress or altered metabolic demand.

Taken together, the structural diversity of ceramides and the compartmentalized organization of their biosynthetic pathways provide the foundation for their functional heterogeneity. Because these pathways are differentially engaged by nutrient availability, inflammatory signaling, and cellular stress, dysregulated ceramide biogenesis is particularly relevant to the development of cardiac pathology.

## 3. Physiological Roles of Ceramides in the Heart

### 3.1. Membrane Organization and Signaling Platforms

Ceramides serve as central regulatory lipids within cells and participate in multiple physiological processes through both structural and signaling mechanisms. A key aspect of ceramide function arises from its ability to modulate membrane organization [7,22]. Because of their small polar headgroup and long hydrophobic chains, ceramides promote tight lipid packing within bilayers and facilitate the formation of ceramide-enriched membrane platforms. These specialized membrane domains organize signaling complexes by promoting clustering of receptors and associated adapter proteins. For example, ceramide accumulation in the plasma membrane promotes clustering of membrane receptors such as tumor necrosis factor receptor-1 (TNFR1) and the Fas/CD95 receptor, thereby enabling efficient assembly of downstream death-inducing signaling complexes [42,43,44]. Although much of this evidence derives from broader cell biology rather than from myocardium specifically, these membrane-organizing properties provide an important foundation for understanding how ceramides influence stress-responsive signaling in cardiac cells.

### 3.2. Ceramide-Binding Proteins and Intracellular Signaling

Beyond membrane organization, ceramides directly regulate intracellular signaling through interactions with specific protein targets, referred to as ceramide-binding proteins. One of the best characterized interactions is with protein phosphatase 2A (PP2A), a serine/threonine phosphatase that can be activated by ceramide binding. Activation of PP2A alters the phosphorylation state of multiple signaling proteins involved in cellular growth and metabolic regulation. Ceramides have also been shown to interact with atypical protein kinase C isoforms, particularly PKCζ, which functions in pathways controlling cell polarity, cytoskeletal organization, and metabolic signaling [45,46,47]. Through these interactions, ceramides act not only as membrane lipids but also as intracellular signaling regulators capable of shaping cellular stress responses.

### 3.3. Trafficking, Exosomes, and the Sphingolipid Rheostat

Ceramides also contribute to intracellular trafficking and membrane dynamics. Their ability to induce negative membrane curvature facilitates membrane budding and vesicle formation within endosomal compartments. This mechanism has been demonstrated during multivesicular body formation, where ceramide generation promotes inward budding of endosomal membranes and the formation of exosomes that participate in intercellular communication [48]. In addition, ceramides occupy a central position within the sphingolipid metabolic network and act as precursors for other bioactive lipid mediators, such as S1P. S1P is a signaling lipid with functions often distinct from those of ceramides, including regulation of vascular integrity, immune cell trafficking, and endothelial function [7,49]. Alternatively, ceramides are converted in the GA into complex sphingolipids, including sphingomyelin or glycosphingolipids through the addition of polar headgroups. These reversible metabolic conversions create a dynamic network often referred to as the “sphingolipid rheostat”, in which the relative balance between ceramide, sphingosine, and S1P influences key cellular processes including proliferation, survival, differentiation and stress responses [49,50]. Together, these roles in membrane organization, intracellular trafficking, and regulated signaling position ceramides as important coordinators of cellular homeostasis under physiological conditions.

### 3.4. Circulating Ceramides Under Physiological Conditions

In addition to intracellular ceramide pools, ceramides are also present in the circulation, where they are transported between organs and contribute to systemic lipid homeostasis. The liver is considered the major source of circulating ceramides under physiological conditions. Hepatocytes actively synthesize ceramides intracellularly and incorporate them into lipoprotein particles during lipoprotein assembly. Consequently, ceramides are transported in the bloodstream within lipoprotein particles including VLDL, LDL, and high-density lipoproteins (HDL) [12,51,52]. This physiological transport function provides an important bridge to later sections, where circulating ceramides are considered in the context of cardiovascular risk, vascular dysfunction, and HF progression [53].

Together, through their roles in membrane organization, intracellular trafficking, regulated signaling, and systemic transport, ceramides contribute to the coordinated functioning of cells and tissues. These physiological functions explain why disturbance of ceramide homeostasis leads to pathological consequences in the heart and the cardiovascular system.

## 4. Ceramides in Human Cardiovascular Disease and Heart Failure

### 4.1. Circulating Ceramides as Biomarkers of Cardiovascular Risk

Human studies have established circulating ceramides as clinically informative lipid biomarkers associated with cardiovascular risk, HF severity, and adverse outcomes. In patients with HF, elevated plasma ceramide levels have been reported across chronic, ischemic, and metabolic forms of disease, where they correlate with disease severity and independently predict mortality, particularly in the setting of impaired systolic function [16]. Beyond overt HF, large observational cohorts have shown that several long-chain and very-long-chain ceramide species, including C16:0, C18:0, and C24:1 ceramides, are robustly associated with increased risk of major adverse cardiovascular events, even after adjustment for conventional risk factors [53,54,55]. These findings indicate that circulating ceramides capture pathophysiological information not fully reflected by standard clinical biomarkers or by traditional lipid measures alone.

This clinical relevance has supported the development of ceramide-based risk algorithms, including Cardiovascular Event Risk Test 1 (CERT1), which further strengthens the biomarker utility of circulating ceramide profiling in cardiovascular disease and HF [56]. In parallel, human lipidomic studies suggest that ceramide dysregulation may be especially relevant in cardiometabolic settings such as obesity, insulin resistance, and diabetes, conditions that substantially increase the risk of cardiomyopathy and HF [57]. Together, these data support the view that circulating ceramides are not merely correlative metabolic by-products, but candidate mechanism-linked biomarkers that reflect broader cardiovascular and metabolic stress.

### 4.2. Lipoprotein Transport and Vascular Effects of Circulating Ceramides

In the circulation, ceramides are distributed across major lipoprotein classes, including HDL, LDL, and VLDL, linking systemic sphingolipid metabolism to vascular and myocardial disease. Plasma ceramide concentrations are dynamically modified by interventions that alter lipoprotein metabolism, including proprotein convertase subtilisin/kexin type 9 (PCSK9) inhibition, statin treatment, weight loss, and metformin therapy [54,58,59,60,61], indicating that circulating ceramide pools are both responsive and potentially modifiable in human disease.

As bioactive lipids carried within lipoproteins, circulating ceramides have also been implicated in vascular pathology. Elevated serum ceramides are strongly associated with coronary artery disease and adverse cardiovascular outcomes, and ceramides accumulate within atherosclerotic plaques, where they are linked to plaque instability [53,62,63]. Mechanistically, ceramides promote endothelial oxidative stress, stimulate superoxide generation, reduce nitric oxide bioavailability, and impair endothelium-dependent vasodilation [64,65,66]. They also amplify inflammatory signaling, including pathways involving tumor necrosis factor-alpha (TNF-α), and can promote LDL aggregation and macrophage uptake, thereby accelerating foam cell formation and atherogenesis [67,68,69,70,71]. These observations suggest that altered systemic ceramide metabolism may not only be a marker for vascular disease but also contribute to a pro-inflammatory and pro-atherogenic environment that favors cardiovascular injury and may secondarily promote myocardial ceramide accumulation during HF development.

### 4.3. Genetic Regulation of Circulating Ceramide Profiles

Genetic studies provide further support for the biological relevance of circulating ceramide profiles in humans. Genome-wide association studies (GWAS) have identified multiple loci associated with plasma sphingolipid levels, including variants in *SPTLC3*, *LASS4*/*CERS4*, and *SGPP1*, all of which encode core components of ceramide synthesis or broader sphingolipid metabolism [72]. Pathway enrichment analyses have shown significant overrepresentation of genes involved in sphingolipid metabolic processes, reinforcing the coherence of these associations at the systems level, particularly within gene regulatory and signaling network frameworks [72]. Family-based analyses further indicate moderate heritability for individual sphingolipid species [73], suggesting that inter-individual variation in circulating ceramide composition is substantially influenced by genetic factors. Independent metabolomic GWAS have replicated associations near *SPTLC3*, confirming a central role for SPT in regulating human sphingolipid levels [74]. Importantly, several ceramide-associated loci overlap with regions of established cardiometabolic risk, implying partial genetic convergence between sphingolipid metabolism and cardiovascular disease susceptibility [75]. Thus, human genetic data supports the concept that circulating ceramide profiles are biologically regulated traits rather than nonspecific consequences of disease.

### 4.4. Myocardial Ceramide Accumulation in Human Heart Failure

In addition to circulating abnormalities, evidence from human cardiac tissue indicates that ceramide accumulation is a consistent feature of the failing myocardium. Analyses of myocardial biopsy samples have demonstrated increased ceramide content in failing human hearts together with upregulation of ceramide biosynthetic pathways [76]. Lipidomic profiling further suggests that these changes do not reflect a uniform elevation in all ceramide species, but rather selective enrichment of defined myocardial ceramide species, including increases in C16:1, C16:0, and C24:1 ceramides [76]. These findings support the view that HF is associated with remodeling of the myocardial sphingolipid profile rather than simple global lipid accumulation.

Myocardial ceramide levels also appear to be dynamically regulated in humans. In advanced heart failure with reduced ejection fraction (HFrEF), myocardial samples obtained at the time of ventricular assist device implantation show markedly elevated ceramide levels compared with non-failing hearts, whereas mechanical unloading through left ventricular assist device (VAD) support is associated with substantial reductions in myocardial ceramides across multiple acyl-chain lengths [76]. This reversibility suggests that myocardial ceramide enrichment is at least partly stress-responsive and may track with hemodynamic burden and contractile dysfunction. By contrast, findings in heart failure with preserved ejection fraction (HFpEF) raise the possibility that circulating and myocardial ceramide pools do not always change in parallel. In HFpEF, metabolic interventions such as gastric bypass surgery have been associated with improved plasma ceramide profiles and enhanced cardiac function, while myocardial ceramide content appears comparatively unchanged [77], though direct myocardial data remain limited. Taken together, these observations suggest that myocardial ceramide accumulation may be more closely linked to contractile dysfunction in HFrEF, whereas circulating ceramide alterations in HFpEF may more strongly reflect systemic metabolic disturbance.

### 4.5. Species-Specific Ceramide Signatures Across Cardiovascular Disease

An important theme emerging from human studies is that the pathological relevance of ceramides depends on molecular species composition rather than total ceramide abundance alone [78,79,80]. Across coronary artery disease, acute coronary syndromes, hypertension, and chronic HF, recurrent enrichment of C16:0-, C18:0-, and C24:1-containing ceramides has been observed [78,81,82,83]. These overlapping species-level patterns across distinct cardiovascular contexts suggest that a relatively conserved subset of ceramide species may underline diverse forms of cardiovascular pathology.

Not all ceramide species, however, appear to confer equivalent risk. Prospective cohort data indicate that very-long-chain saturated ceramides, particularly Cer-22 and Cer-24 species, may be associated with more favorable cardiovascular outcomes in some settings [80]. In the Cardiovascular Health Study, higher plasma Cer-22 levels were independently associated with lower HF incidence, while higher Cer-22/Cer-16 and Cer-24/Cer-16 ratios were associated with lower risk in both HFrEF and HFpEF [80]. Other studies and meta-analytic data similarly suggest that ratios involving long-chain ceramides may correlate more strongly with adverse outcomes than ratios dominated by very-long-chain species. In particular, a meta-analysis of prospective clinical studies reported that ceramide ratios such as C18:1/C16:0 and C18:1/C18:0 were more strongly associated with negative cardiovascular outcomes than the very-long-chain ratio C18:1/C24:0 [53]. Collectively, these findings indicate that species- and chain-length-specific analyses are essential for interpreting ceramide-related cardiovascular risk, as well as support the view that ceramides function not only as clinically informative biomarkers but also as candidate mediators linking systemic metabolic dysfunction to myocardial remodeling and HF progression (Table 1).

## 5. Ceramide Accumulation in Pre-Clinical Models of Heart Failure

### 5.1. Overview of Experimental Evidence

While human cohort studies establish robust associations between circulating ceramide species and cardiovascular risk, these observational data cannot fully resolve causality or identify tissue-specific mechanisms. Pre-clinical animal models therefore provide proof-of-concept evidence that ceramide accumulation in the myocardium is associated with cardiac pathology. Across a broad range of experimental settings, including metabolic, ischemic, and genetically manipulated models, increases in myocardial ceramide levels have been reported in parallel with the development of cardiac dysfunction [15,76,88,89]. Together, these findings suggest that ceramide accumulation frequently accompanies pathological cardiac remodeling across diverse HF-relevant contexts.

### 5.2. Metabolic Disease Models

In rodent models of diabetes and obesity, myocardial lipid overload develops alongside impaired cardiac performance, with ceramides emerging as prominent toxic lipid intermediates. In Akita Ins2 (WT/C96Y) mice, a genetic model of type 1 diabetes, myocardial ceramide levels are elevated and associated with the development of diastolic dysfunction [88]. Similarly, Zucker diabetic fatty (ZDF) rats, which exhibit obesity, insulin resistance, and hyperlipidemia, display increased myocardial ceramide content together with impaired ventricular relaxation and diastolic dysfunction [90]. These studies indicate that ceramide accumulation parallels the emergence of cardiac dysfunction in settings of chronic diabetic and obesity-related stress.

### 5.3. Diet-Induced Lipid Overload and Ceramide Remodeling

Diet-induced metabolic models further support the link between lipid oversupply, ceramide accumulation, and cardiac dysfunction. When fatty acid uptake exceeds oxidative capacity, excess lipid is diverted toward storage and biosynthetic pathways, including de novo ceramide synthesis [5]. Although quantitatively minor relative to total lipid flux, this pathway appears to be preferentially engaged under conditions of lipid oversupply and may exert disproportionate pathological effects. In mouse models fed diets enriched in saturated fatty acids, pathological cardiac hypertrophy and functional impairment develop in parallel with activation of sphingolipid biosynthetic pathways and lipidomic remodeling of myocardial ceramide species, including increases in long-chain ceramides [91,92,93]. These findings support the concept that nutrient excess drives pathogenic sphingolipid remodeling in the heart.

### 5.4. Ischemia–Reperfusion Injury

Beyond chronic metabolic overload, ischemia–reperfusion (I/R) injury represents a major pathological context in which myocardial ceramide accumulation has been consistently observed. In rat models of left coronary artery occlusion, myocardial ceramide content increased to approximately 155% during early ischemia and further rose to 250% following 3 h of reperfusion, indicating progressive accumulation during the reperfusion phase [94,95]. Similar ceramide responses have been reported across multiple organ I/R models [96,97,98], suggesting that ceramide elevation represents a conserved cellular stress response rather than a phenomenon restricted to chronic metabolic disease.

Mechanistically, both sphingomyelin hydrolysis and de novo synthesis appear to contribute to the phenomenon. Activation of neutral and acid sphingomyelinases during I/R promotes rapid hydrolysis of membrane sphingomyelin, generating ceramide during the early phases of injury [99,100], while later reperfusion is also associated with increased activity of the de novo pathway, including upregulation of SPT [101]. In parallel, oxygen availability directly influences ceramide metabolism during ischemia. DEGS1, the oxygen-dependent enzyme that converts dihydroceramide to ceramide, is inhibited under hypoxic conditions, leading to accumulation of dihydroceramide species that have also been associated with exacerbation of I/R injury [102]. Collectively, these findings indicate that ceramide elevation is a dynamic and stress-responsive feature of myocardial injury.

All findings indicate that ceramide accumulation is reproducibly observed across multiple HF-relevant settings, including chronic metabolic stress, diet-induced lipid overload, and acute ischemic injury. These models therefore provide critical support for the concept that ceramide dysregulation is not merely a correlative feature of human disease, but a recurring component of cardiac pathological remodeling that warrants direct mechanistic interrogation.

## 6. Pharmacological and Genetic Modulation of Ceramide Biosynthesis

### 6.1. Evidence from Animal Models

Pharmacological and genetic studies in animal models have substantially strengthened the view that ceramide accumulation is mechanistically involved in cardiometabolic pathology rather than merely associated with it. One of the most widely studied approaches has been inhibition of ceramide de novo synthesis through blockade of SPT, the rate-limiting enzyme in the pathway. Myriocin, a potent SPT inhibitor, has consistently improved systemic metabolic phenotypes in preclinical models, including improving glucose tolerance, reducing insulin resistance, and preventing or reversing obesity-related metabolic dysfunction. In ZDF rats, *db/db* mice, and high-fat-diet insulin-resistant models, myriocin lowers ceramide levels and significantly improves insulin sensitivity, including marked reductions in the HOMA-IR index [103,104,105]. These findings indicate that suppressing de novo synthesis can broadly reshape systemic metabolic homeostasis.

Genetic evidence supports a similar conclusion. Deletion of *Cers6*, the enzyme responsible for C16:0 ceramide synthesis, protects mice from diet-induced insulin resistance and steatohepatitis [57], indicating that individual ceramide synthase isoforms can exert species-specific pathological effects under metabolic stress. Partial genetic reduction in SPT activity in *Sptlc1* haplo-insufficient mice also decreases intestinal cholesterol absorption and lowers circulating cholesterol [106], further supporting the concept that inhibition of excessive sphingolipid synthesis has broad metabolic consequences extending beyond the heart. Together, these systemic models indicate that reducing ceramide synthesis modifies disease-relevant metabolic phenotypes rather than simply altering lipid composition in a neutral manner.

#### 6.1.1. Cardiac Injury and Lipotoxic Cardiomyopathy

Evidence from cardiac injury models further supports a causative role for ceramide overproduction in myocardial damage. In mice subjected to myocardial I/R injury, intraventricular administration of myriocin reduced infarct size and attenuated cardiac hypertrophy. These protective effects were associated with increased expression of transcription factor EF (TFEB) and peroxisome proliferator-activated receptors (PPARs), accompanied with enhanced mitochondrial fatty acid import and beta-oxidation and improved energy utilization during post-ischemic recovery [107]. In a related post-conditioning setting, myriocin also increased activation of the nuclear factor erythroid 2-related factor 2 (Nrf2) and heme oxygenase-1 (HO)-1 signaling pathway, reduced infarct area by approximately 40.9%, and promoted antioxidant response in murine models [101], supporting the view that reduced ceramide accumulation can modify downstream injury signaling in the heart.

Additional evidence comes from lipotoxic cardiomyopathy models. In glycosylphosphatidylinositol-anchored lipoprotein lipase (LpL) overexpressing transgenic mice fed with a chow diet, myriocin treatment rescued cardiac function and significantly reduced mortality rates [108]. In this model, inhibition of ceramide overproduction preserved systolic function, and improved survival, supporting a critical role for toxic lipid intermediates in the pathogenesis of lipotoxic cardiomyopathy [108]. Downstream targeting of ceramide synthesis has also shown benefit. In a murine model of I/R, inhibition of DES1 with the small-molecule inhibitor CIN038 increased dihydroceramides at the expense of ceramides and has been reported to attenuate hypertrophy and fibrosis while improving mitochondrial indices [109,110,111]. Collectively, these findings indicate that pharmacological suppression of ceramide excessive synthesis alleviates myocardial injury and remodeling phenotypes, thereby further supporting a causal role for ceramide accumulation in cardiac pathology.

#### 6.1.2. Genetic Evidence in Animal Models

Genetic manipulation of ceramide metabolic enzymes has provided more direct in vivo support for causal involvement of ceramide synthesis in HF progression. In cardiomyocyte-specific *Sptlc2* knockout mice, myocardial ceramide levels were significantly reduced, and post-infarction cardiac dysfunction was attenuated. In this model, total myocardial ceramides was decreased by approximately 31%, systolic impairment was alleviated, and ventricular dilatation trended toward improvement [76], indicating that controlling endogenous ceramide de novo synthesis within cardiomyocytes can mitigate adverse remodeling after myocardial injury.

Genetic disruption of downstream enzymes within the de novo pathway provides additional mechanistic support. In mouse models of obesity and diet-induced metabolic dysfunction, genetic ablation of *Degs1*, which catalyzes the conversion of dihydroceramide to ceramide, reduces ceramide/dihydroceramide ratios, resolves hepatic steatosis, and improves insulin sensitivity [112]. Although direct HF phenotypes linked to *Degs1* remain less well defined, evidence from murine hypoxia models indicates that *Degs1* is dynamically regulated in the heart, where reduced expression is associated with decreased ceramide and accumulation of dihydroceramide [113]. These findings suggest that the desaturation step may function as a stress-responsive checkpoint within cardiac ceramide metabolism.

Upstream lipid regulatory pathways also influence ceramide biosynthesis. Stearoyl-CoA desaturase-1 (SCD1), a key determinant of monounsaturated fatty acid production, modulates substrate availability for ceramide synthesis. SCD1-deficient mice exhibit reduced monounsaturated ceramide synthesis together with suppression of SPT activity, and lipidomic analyses reveal substantial remodeling of ceramide species composition [114,115]. These findings expand the regulatory framework of ceramide biology beyond core synthetic enzymes and indicate that altered fatty acid desaturation can indirectly reshape myocardial sphingolipid profiles.

### 6.2. Evidence from Cellular Models

#### 6.2.1. Increased De Novo Synthesis in Cardiomyocyte-like Cells

Cellular studies provide complementary mechanistic support for the pathogenic role of ceramide synthesis by allowing direct manipulation of individual enzymes in cardiomyocyte-like systems. In AC16 cardiomyocyte-like cells, overexpression of *SPTLC1* or *SPTLC2* induces robust accumulation of total and selected long- and very-long-chain ceramide species, accompanied by increased apoptosis and impaired oxidative metabolism, including reduced basal respiration and respiratory capacity [76]. These findings indicate that exaggerated ceramide de novo synthesis is sufficient to induce a pro-death, metabolically compromised phenotype in cardiomyocyte-like cells.

#### 6.2.2. Species-Specific Synthase Function in Cellular Models

Other cellular models highlight the importance of ceramide synthase isoform specificity. In cardiomyocyte models of acute hypoxia, accumulation of very-long-chain dihydroceramides has been linked to increased expression of CerS2, and elevated very-long-chain (VLC) dihydroceramides have also been associated with arrhythmias and HF in human myocardial biopsies. Consistent with this, *Cers2* knockdown in HL-1 cardiomyocytes reduces VLC-dihydroceramide abundance and alters regulators of Ca^2+^ handling and electrical conduction [87]. siRNA-mediated isoform-specific silencing studies further support divergent roles of ceramide synthases in cardiac remodeling: suppression of CerS2 exacerbates hypertrophic responses in human cardiomyocytes, whereas blockage of CerS5 and CerS6 produces a comparatively protective phenotype [116]. Together, these cellular observations suggest that distinct ceramide synthase isoforms, and the species they generate, differentially regulate maladaptive remodeling through chain-length-specific sphingolipid profiles.

Taken together, evidence from both animal and cellular models moves ceramide from association toward causation (Table 2), while also showing that the pathological consequences of ceramide accumulation depend on biosynthetic context, enzyme specificity, and species composition.

## 7. Ceramide-Induced Mitochondrial Dysfunction in Heart Failure

### 7.1. Ceramide Accumulation as a Driver of Metabolic Dysfunction in Failing Myocardium

In failing myocardium, ceramide accumulation appears to reflect more than a passive metabolic by-product of disturbed lipid handling. The failing heart develops defects in both fatty acid and glucose utilization, accompanied by mitochondrial dysfunction, reduced oxidative metabolism, and accumulation of toxic lipid intermediates, one of which is ceramide [76,84,117,118]. Consistent with a pathogenic role, abnormal ceramide biosynthesis and accumulation have been linked to myocardial injury and HF progression [108], while aberrant de novo ceramide synthesis has been associated with progressive pathological cardiac remodeling and dysfunction [76].

Ceramides may also interact with systemic metabolic regulators that influence cardiac bioenergetics. Ghrelin, a stomach-derived hormone that stimulates appetite, has been shown to promote fatty acid oxidation and suppress glucose utilization in the failing heart. Importantly, ghrelin’s metabolic actions are mediated, at least in part, by intracellular ceramide signaling regulated via carnitine palmitoyltransferase 1C (CPT1C) [119,120]. Although this interaction has not been fully characterized in cardiomyocytes, it suggests that ceramides may serve as key intermediates linking hormonal signals to mitochondrial substrate selection and energy metabolism.

These observations support the fact that ceramides are not only markers of lipotoxic in the myocardium, but also candidate mediators of the metabolic and structural abnormalities that drive HF progression.

### 7.2. Mitochondrial Bioenergetics and Membrane Permeabilization

Mitochondria represent a major downstream target of ceramide-mediated lipotoxicity. Mechanistic studies indicate that ceramides directly disrupt mitochondrial homeostasis through multiple pathways, including impaired respiration, reduced oxidative capacity, and increased oxidative stress [84,117,118]. Consistent with a direct bioenergetic effect, exogenous ceramide has been shown to acutely inhibit adenosine diphosphate (ADP)-stimulated mitochondrial respiration in isolated heart mitochondria, and cell-permeable ceramide has further been reported to directly inhibit mitochondrial respiratory chain Complex III, providing a more specific mechanistic basis for ceramide-induced impairment of oxidative phosphorylation [121]. In AC16 human cardiomyocyte-like cells, dysregulated de novo ceramide synthesis through *SPTLC1* or *SPTLC2* overexpression was associated with reduced basal respiration and respiratory capacity, together with increased apoptosis, supporting a direct link between ceramide synthesis and mitochondrial dysfunction [76].

In addition to impairing mitochondrial bioenergetics, ceramides can disrupt mitochondrial membrane integrity at multiple levels. Accumulation within the inner mitochondrial membrane is likely to interfere with oxidative phosphorylation, whereas enrichment in the outer mitochondrial membrane can increase permeability by interacting with mitochondrial membrane proteins such as the voltage-dependent anion channel (VDAC) or by self-assembling into large membrane channels [85,86]. In line with this, exogenous C2- and C16-ceramides have been reported to induce cytochrome c release from isolated mitochondria [122,123,124], while complementary biophysical studies suggest that ceramides are capable of forming large pores in phospholipid membranes, providing a plausible mechanism for outer membrane permeabilization [125]. Although much of this evidence derives from reductionist experimental systems, it supports a mechanistic framework in which ceramide accumulation promotes cytochrome c release, apoptotic signaling, and mitochondrial damage. Ceramides have also been implicated in mitochondrial quality control, as C18-ceramide generated by CerS1 can bind microtubule-associated protein 1 light chain 3B (LC3B)-II on mitochondrial membranes and promote dynamin-related protein 1 (Drp1)-dependent targeting of autophago-lysosomes to mitochondria, leading to lethal mitophagy and inhibition of mitochondrial oxygen consumption. Together, these findings suggest that ceramides compromise mitochondrial homeostasis through multiple complementary mechanisms, including membrane permeabilization and mitophagy-associated mitochondrial clearance, although the relevance of some of these pathways in cardiomyocytes remains to be fully established.

### 7.3. Mitochondrial Dynamics, MAMs, and Calcium Handling

Beyond their effects on mitochondrial bioenergetics and membrane permeabilization, ceramide accumulation has also been linked to dysregulation of mitochondrial dynamics, including enhanced fission and fragmentation. Such changes may further exacerbate mitochondrial dysfunction by destabilizing network integrity and reducing the capacity of cardiomyocytes to adapt to metabolic stress. Notably, C16-ceramide generated by CerS6 has been shown to bind mitochondrial fission factor (Mff), promoting Drp1 recruitment and mitochondrial network fragmentation [84]. These observations suggest that ceramide species have distinct effects on mitochondrial morphology in a chain-length-specific manner, thereby further impairing cellular stress adaptation.

Ceramides may also affect inter-organelle communication at mitochondria-associated membranes (MAMs), which serve as critical hubs for calcium transfer between the endoplasmic reticulum and mitochondria. Because mitochondrial calcium overload is a key trigger for mitochondrial permeability transition pore (mPTP) opening, ceramide accumulation at MAMs has been proposed to amplify mitochondrial stress responses by altering calcium signaling and ER–mitochondrial crosstalk [126]. Although this mechanism has not been directly demonstrated in cardiomyocytes, it provides a plausible framework linking ceramide accumulation to altered bioenergetic efficiency and increased susceptibility to stress. Thus, alongside direct effects on respiration and membrane integrity, ceramides may also promote HF progression by destabilizing mitochondrial dynamics and calcium handling.

## 8. Therapeutic Targeting of Ceramide Metabolism in the Heart

### 8.1. Direct Targeting of Ceramide Synthesis

Among current therapeutic approaches, direct inhibition of ceramide synthesis provides the clearest proof-of-concept for targeting ceramide-driven pathology. The best-studied strategy so far targets SPT. In experimental models, myriocin has shown beneficial effects across metabolic dysfunction, myocardial injury, and lipotoxic cardiomyopathy, supporting this pathway as a tractable point of intervention [108]. However, because SPT lies upstream of the broader sphingolipid network, sustained inhibition is likely to alter either normal ceramide dynamics or multiple downstream metabolites beyond ceramide itself. Although myriocin has been highly valuable in demonstrating that reducing ceramide overproduction can improve cardiac and metabolic phenotypes, its broad pathway effects may limit selectivity and translational suitability.

More selective intervention may target the downstream enzymes. CerSs and DES1 represent attractive alternative targets because they may allow narrower modulation of ceramide production and species composition. Fumonisin B1, a mycotoxin that competitively inhibits CerSs has been useful mechanistically as a CerS inhibitor, but its hepatotoxicity and nephrotoxicity make it unsuitable as a therapeutic candidate [127,128]. By contrast, DES1-directed strategies have shown more promising translational potential. Small-molecule inhibition with CIN038 attenuated ceramide-driven responses in cardiac models, while other DES1-modulating approaches such as fenretinide have shown metabolically beneficial effects in non-cardiac settings [110,129]. Together, these observations suggest that downstream modulation of ceramide species may preserve some therapeutic benefit while avoiding part of the broader pathway suppression associated with upstream inhibition.

### 8.2. Indirect Modulation of the Sphingolipid Network

In addition to direct inhibition of ceramide synthesis, indirect modulation of the wider sphingolipid network may also offer therapeutic potential. One such approach involves shifting the ceramide-sphingosine-S1P balance. FTY720 (fingolimod), a sphingosine analogue that acts primarily through S1P-related signaling after phosphorylation, has shown cardioprotective effects in models of hypertrophy, myocardial infarction, and cardiac transplantation [130,131]. These findings suggest that broader sphingolipid pathway modulation can influence cardiac remodeling, stress signaling, and survival pathways. However, because the actions of FTY720 are pleiotropic and extend well beyond ceramide biology, its relevance here is mainly conceptual rather than ceramide-specific [132].

A second indirect strategy targets stress-induced ceramide generation through sphingomyelin hydrolysis. Both ASMases and nSMases have been explored pharmacologically, including with functional inhibitors of acid sphingomyelinase (FIASMAs) such as amitriptyline and with the nSMase inhibitor GW4869 [133,134,135]. Available preclinical data suggest that these approaches reduce stress-upregulated ceramide production and confer cardioprotective effects in injury settings. However, evidence in the heart remains limited, and most studies are exploratory rather than translational. Nevertheless, these findings expand the therapeutic landscape by demonstrating that intracellular ceramide homeostasis can be modulated not only through de novo synthesis, but also via stress-responsive membrane pathways.

### 8.3. Translational Challenges and Future Therapeutic Considerations

Despite growing experimental evident supporting the therapeutic potential of targeting ceramide metabolism, solid findings remain preclinical. The currently discussed strategies are not yet established as a ceramide-related therapy in routine cardiovascular practice, although some relevant compounds are already used clinically in other contexts, such as FTY720 or certain FIASMAs [132,134].

Several issues limiting translational implications need to be addressed in future. First, ceramide metabolism is tightly integrated with the broader sphingolipid network, so intervention at a single enzymatic step may produce wide-ranging downstream effects. Second, individual ceramide species differ in chain length, biosynthetic origin, and biological function, raising the possibility that nonspecific lowering of total ceramide synthesis may obscure both harmful and potentially adaptive ceramide pools. Third, tissue specificity remains unresolved: systemic modulation of ceramide metabolism may improve whole-body metabolic phenotypes, but it is still unclear whether pathogenic myocardial ceramide pools can be selectively targeted without disrupting ceramide-dependent biological functions in other organs. Future therapeutic development will therefore require greater precision, including species-selective, tissue-selective, and compartment-specific approaches, together with tighter integration of human lipidomic profiling and mechanistic cardiac studies. In this sense, the key translational goal is no longer simply to reduce ceramides globally, but to define which ceramide pools are pathogenic, in which cardiac contexts, and at what stage of disease they are most therapeutically tractable; thus, an advanced understanding of the molecular mechanisms underlying ceramide biosynthesis under physiological and pathological conditions is required.

## 9. Conclusions and Future Perspectives

Ceramides are central intermediates within the sphingolipid metabolic network and play important roles in membrane organization, lipid signaling, and metabolic regulation. Across the sections of this review, emerging evidence from human lipidomics, myocardial tissue analyses, animal models, and cellular studies converge on the view that dysregulated ceramide metabolism contributes to cardiovascular pathology, including the development and progression of HF. Both circulating ceramides derived from peripheral metabolic tissues and ceramides generated locally within cardiac cells are relevant to myocardial stress responses and pathological remodeling. Clinical lipidomic studies have consistently demonstrated that specific circulating ceramide species and ratios are strongly associated with adverse cardiovascular events, highlighting their potential utility as biomarkers for cardiovascular risk stratification. Furthermore, growing evidence suggests that ceramides should not be regarded solely as markers of disease, but also as candidate mediators of cardiac lipotoxicity, metabolic dysfunction, and HF progression.

Despite these advances, several important questions remain unresolved. Direct evidence examining ceramide accumulation and metabolism in human cardiac tissue is still limited, particularly in comparison with the much larger body of circulating lipidomic data. In addition, the molecular mechanisms through which ceramides impair cardiomyocyte function, especially in relation to mitochondrial or other organelle dysfunction, membrane organization and stress signaling, are not yet fully understood in human-relevant settings. Another key uncertainty is the relationship between circulating and myocardial ceramide pools. A limitation in the current field is the incomplete understanding of the relative effects of extracellular versus intracellular ceramide pools. Although circulating ceramides are strongly associated with cardiovascular risk and HF outcomes, it remains unclear whether extracellular ceramides directly exert pathogenic effects on cardiac cells or primarily reflect systemic metabolic stress. Likewise, intracellular ceramide accumulation may arise from cardiomyocyte-intrinsic synthesis, sphingomyelin hydrolysis, salvage pathways, uptake from extracellular sources, or a combination of these processes. Available human data suggests that these compartments may not always change in parallel across HF phenotypes, raising the possibility that HFrEF and HFpEF differ in the extent to which local versus systemic ceramide dysregulation predominates. Species specificity represents a further major challenge, as growing evidence indicates that chain length, saturation, and biosynthetic origin all shape the biological effects of ceramides, making total ceramide burden alone an insufficient descriptor of pathogenic relevance.

Therapeutic targeting of ceramide metabolism is promising but remains largely underexplored in clinical settings. Pharmacological inhibition of de novo excessive synthesis, modulation of downstream enzymes, and targeting of sphingomyelin hydrolysis have all shown cardioprotective or metabolically beneficial effects in experimental models. However, translation into clinical therapies remains challenging due to issues of dosing and treatment duration, pathway complexity, tissue-specific effects, and the diverse biological roles of individual ceramide species. Future therapeutic development will need to address not only efficacy, but also selectivity, safety, and the risk of disrupting broader sphingolipid homeostasis. Moreover, our current understanding of the regulatory mechanisms governing ceramide biogenesis, compartmentalization, and species-specific effects remains incomplete, particularly in the human heart. This is further compounded by the dual nature of ceramides as both structural and membrane components and signaling molecules, meaning that global inhibition of ceramide synthesis is unlikely to be viable without disrupting essential cellular functions. In addition, the biological effects of ceramides are highly dependent on acyl-chain composition and subcellular localization; however, it remains unclear which specific ceramide species are causally detrimental in cardiac disease. This lack of specificity limits the development of targeted interventions. Moreover, current pharmacological approaches lack the ability to selectively modulate ceramide metabolism in a tissue- or cell type-specific manner, raising concerns regarding off-target effects in other metabolically active organs. Technical challenges in accurately quantifying and distinguishing individual ceramide species also complicate their application as reliable clinical biomarkers. Together, these limitations highlight the need for a more refined understanding of ceramide biology and the development of more selective and targeted strategies before clinical translation can be realized.

An important priority for future studies is to determine whether ceramides should be viewed primarily as biomarkers of cardiovascular risk or as modifiable mediators of disease progression. Although current human evidence more strongly supports their role as clinically informative biomarkers, accumulating mechanistic and preclinical intervention studies suggest that ceramides may also actively contribute to pathological remodeling. Addressing this question will require closer integration of human lipidomic profiling, longitudinal outcome studies, and mechanistic experiments designed to test whether modulation of specific ceramide pools can alter disease trajectory.

Future studies should therefore focus on several perspectives. Firstly, the relative contribution of systemic versus cardiac-intrinsic ceramide production to myocardial ceramide accumulation during HF needs to be clarified. Secondly, further studies combining circulating lipidomic profiling with direct myocardial analyses will be essential to determine whether cardiac ceramides can enter the circulation and serve as potential biomarkers of myocardial stress, injury, or the susceptibility to HF. Third, mechanistic work should move beyond total ceramide measurements toward species-selective and compartment-specific analyses that can better define which ceramide pools are pathogenic in different disease contexts. Such findings may also help personalize toxic lipid-related treatment, through more precise mechanism-based care. Finally, integrating human lipidomic datasets with mechanistic animal and cellular models will be critical for determining how modulation of ceramide metabolism can be translated into effective diagnostic and therapeutic strategies for cardiovascular disease.

To conclude, ceramides are increasingly viewed not only as biomarkers of cardiovascular risk, but also as candidate mediators of cardiac lipotoxicity, remodeling, and HF progression. This shift in perspective is supported by observations that the failing myocardium accumulates toxic lipid intermediates alongside impaired substrate utilization and mitochondrial dysfunction, and by experimental evidence linking altered ceramide synthesis to pathological cardiac remodeling. However, several important questions remain unresolved. These include the relative contribution of circulating vs. myocardial ceramide pools to cardiac dysfunction, whether specific ceramide species exert distinct and potentially opposing biological effects, and to what extent modulation of ceramide metabolism can be translated into safe and effective therapeutic strategies.

## Figures and Tables

**Table 1:** Representative species-specific ceramide effects and their functional consequences in the heart.

Ceramide Species/Class	Association or Effect	Functional Consequence	Evidence Context
C16:0 ceramide	Adverse cardiovascular risk; CerS6-derived C16:0 ceramide promotes mitochondrial fission	Adverse cardiovascular outcomes, pathological remodeling, and mitochondrial fragmentation	Human circulating lipidomic studies and mechanistic experimental studies [53,54,55,78,81,82,83,84]
C18:0 ceramide	Increased cardiovascular risk; C18 ceramide has been implicated in mitophagy-related mitochondrial injury	Adverse cardiovascular events and impaired mitochondrial homeostasis	Human circulating studies and mechanistic studies [53,54,55,85,86]
C24:1 ceramide	Adverse cardiovascular risk and enriched in failing myocardium	Maladaptive myocardial sphingolipid remodeling in HF	Human plasma and myocardial lipidomic studies [53,54,55,76]
C16:1 ceramide	Increased level in failing myocardium	Myocardial ceramide remodeling in failing hearts	Human myocardial tissue analysis [76]
C22 ceramide	Lower HF incidence in some cohorts	Potentially protective association in certain HF settings	Prospective human cohort study [80]
C24 ceramide	Higher relative abundance associated with lower risk compared with C16 species	Very-long-chain ceramides may be less harmful or relatively protective in some settings	Human cohort analyses based on ceramide ratios [80]
Very-long-chain dihydroceramides	Increased level under hypoxic stress and linked to electrical remodeling	altered Ca^2+^ handling and conduction-related regulators, with relevance to arrhythmia and HF	HL-1 cardiomyocytes and human myocardial biopsy-linked observations [87]

**Table 2:** Experimental modulation of ceramide biosynthesis in animal and cellular models.

Model	Intervention/Target	Effect on Ceramide Metabolism	Main Outcome
ZDF rats, *db/db* mice, and HFD insulin-resistant models [103,104,105]	Myriocin (SPT inhibitor)	Reduced de novo ceramide synthesis and lowered tissue ceramide levels	Improved glucose tolerance, reduced insulin resistance, and decreased HOMA-IR
Diet-induced metabolic stress models [57]	*Cers6* deletion	Reduced production of C16:0 ceramide	Protection from insulin resistance and steatohepatitis
*Sptlc1* haplo-insufficient mice [106]	Partial genetic reduction in SPT activity	Reduced sphingolipid synthesis	Decreased intestinal cholesterol absorption and reduced circulating cholesterol
Murine myocardial I/R injury [107]	Myriocin (intraventricular administration)	Suppressed de novo ceramide synthesis	Reduced infarct size, attenuated hypertrophy, and improved post-ischemic recovery
Murine I/R post-conditioning model [101]	Myriocin	Reduced ceramide accumulation	Increased Nrf2/HO-1 signaling, enhanced antioxidant responses, and reduced infarct area
Lipotoxic cardiomyopathy model (cardiac LpL-overexpressing mice) [108]	Myriocin	Reduced ceramide overproduction	Preserved systolic function and reduced mortality
Murine I/R model [109,110,111]	DES1 inhibition (e.g., CIN038)	Increased dihydroceramides at the expense of ceramides	Reduced hypertrophy and fibrosis and improved mitochondrial indices
Cardiomyocyte-specific *Sptlc2* knockout mice after MI [76]	Genetic deletion of *Sptlc2*	Reduced myocardial ceramide levels	Attenuated post-infarction dysfunction and improved remodeling
Obesity/diet-induced metabolic dysfunction models [112]	*Degs1* ablation	Reduced ceramide/dihydroceramide ratio	Improved insulin sensitivity and reduced hepatic steatosis
Murine hypoxia models [113]	Altered *Degs1* expression	Decreased ceramide with accumulation of dihydroceramide	Stress-responsive remodeling of cardiac sphingolipid metabolism
*Scd1*-deficient mice [114,115]	Loss of *Scd1*	Reduced monounsaturated ceramide synthesis and suppressed SPT activity	Broad remodeling of ceramide species composition
AC16 cardiomyocyte-like cells [76]	*SPTLC1* or *SPTLC2* overexpression	Increased total, long-chain, and very-long-chain ceramides	Increased apoptosis and reduced basal respiration and respiratory capacity
HL-1 cardiomyocytes under acute hypoxia [87]	CerS2 upregulation or inhibition	CerS2 promoted VLC-dihydroceramide accumulation, whereas knockdown reduced VLC-dihydroceramides	Altered Ca^2+^ handling and electrical conduction regulators
Human cardiomyocytes [116]	Isoform-specific *CERS2*, *CERS5*, and *CERS6* silencing by siRNA	Differential effects on ceramide species depending on the synthase targeted	*CERS2* silencing exacerbated hypertrophy, whereas *CERS5*/*CERS6* silencing was relatively protective

**Abbreviations**: HFD, high-fat diet; HOMA-IR, homeostatic model assessment for insulin resistance; I/R, ischemia/reperfusion; Nrf2, nuclear factor erythroid 2-related factor 2; HO-1, heme oxygenase-1; VLC, very-long-chain; siRNA, small interfering RNA.

## Data Availability

No new data were created or analyzed in this study. All figures were created in BioRender. Fonseka, O. (2026) https://BioRender.com/n4zuo6c.
